# Unipolar magnetic field pulses as an advantageous tool for ultrafast operations in superconducting Josephson “atoms”

**DOI:** 10.3762/bjnano.10.152

**Published:** 2019-07-29

**Authors:** Daria V Popolitova, Nikolay V Klenov, Igor I Soloviev, Sergey V Bakurskiy, Olga V Tikhonova

**Affiliations:** 1 Lomonosov Moscow State University Physics Department, Moscow, 119991, Russia; 2 All-Russian Research Institute of Automatics n.a. N.L. Dukhov (VNIIA), 127055, Moscow, Russia; 3 Moscow Technical University of Communications and Informatics (MTUCI), 111024 Moscow, Russia; 4 Lomonosov Moscow State University Skobeltsyn Institute of Nuclear Physics, Moscow, 119991, Russia

**Keywords:** adiabatic superconducting logic, Josephson “atoms”, quantum-state-control, superconducting qubits, ultrafast quantum operations

## Abstract

A theoretical approach to the consistent full quantum description of the ultrafast population transfer and magnetization reversal in superconducting meta-atoms induced by picosecond unipolar pulses of a magnetic field is developed. A promising scheme based on the regime of stimulated Raman Λ-type transitions between qubit states via upper-lying levels is suggested in order to provide ultrafast quantum operations on the picosecond time scale. The experimental realization of a circuit-on-chip for the discussed ultrafast control is presented.

## Introduction

One of the main problems of modern quantum physics is the transfer and storage of quantum information. This is why the development of quantum logic protocols using the fast state control of promising qubits and registers is of great importance. Superconducting artificial atoms based on Josephson junction circuits underlie a number of developments in algorithmic and adiabatic quantum computers, artificial metamaterials, and quantum neural networks. Hence, they seem to be very promising for studies of novel types of fast quantum-state control or initialization [1–15]. In this work, we pay special attention to the search for fundamental possibilities of increasing the speed of population transitions between two selected (“qubit”) states of a multilevel meta-atom with “controlled” selection rules.

Indeed, in the case when the resonance carrier (“filled”) pulses are used for the qubit state preparation and control, the characteristic Rabi-period appears to be in the nanosecond range, which is caused by the small energy separation and near-degeneracy of the atomic levels. For this reason an important task is to decrease the time of the operations with meta-atoms (qubits, qutrits, quantum neurons) to improve the speed of quantum computations as well as the relationship between decoherence time and characteristic duration of basic logic gates. One of the possible ways to solve this problem for different types of superconducting artificial atoms [5–7] is to use unipolar pulses of a magnetic field with picosecond duration and almost rectangular envelopes. Such pulses seem to be very attractive due to their broad frequency spectrum with pronounced near-zero components. The possibilities to control (as well as to read out the states of) either “charge” (including transmons) or “flux” qubits and to induce the Rabi-oscillations by using trains of such pulses with different repetition period were shown in [16–28]. However, because of the broad spectrum, such unipolar pulses give rise to transitions not only between the two lowest (“qubit”) states but also induce transitions to the upper levels of the effective Hamiltonian of the magnetic meta-atom. As a result, it is difficult to transfer the total population from one qubit state to another and to fully provide the required operations (“Not”, “Hadamar” et cetera) with a fidelity equal to 1.

In this paper we suggest a method for the ultrafast control of the population dynamics and population transfer between the qubit states in superconducting meta-atoms by unipolar pulses using the regime of stimulated Raman Λ-type transitions between them via upper-lying levels. The possibility to perform the simplest “Not”-operation (bit-flip or π-rotation of the Bloch vector of the qubit state around x-axis) on the picosecond scale and even faster is demonstrated. The advantages of unipolar pulses working in the Λ-type configuration in comparison to direct population transitions are established. The experimental realization of the theoretically developed scheme is worked out and described.

This paper is organized as follows. In the next section we present our model and discuss the developed theoretical approach. In the section “Results and Discussion” we firstly show the theoretical results obtained for the scheme with blocked direct transitions between qubit states and demonstrate an ultrafast Raman Λ-type “Not”-operation stimulated by a unipolar magnetic pulse (first subsection). Further, we study the influence of additional upper-lying levels of the studied superconducting meta-atom on the time of the obtained ultrafast qubit control. We include an additional fourth level in the consideration and analyze the possible time of the population transfer. Then we analyze the interaction of the superconducting meta-atom with the unipolar pulse for allowed direct transition between qubit states and show this case to be irrelevant (second subsection) and finally we present the experimental realization of our suggested promising scheme (third subsection). At the end we give the conclusions.

## Model and Theoretical Approach

**Figure 1:**
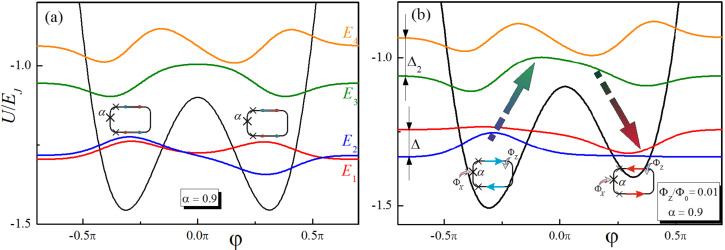
(a) The potential energy and the eigenfunctions (with energies *E*_1_ ,*E*_2_, *E*_3,_ *E*_4_) of the three-junction qubit (described in [3]), where Josephson energies of the elements are *E*_Jos_, *E*_Jos_ and α*E*_Jos_ (α = 0.9, junctions depicted as crosses). The characteristic Josephson energy *E*_Jos_ is 80 times larger than the characteristic Coulomb energy of the heterostructures. φ is the generalized coordinate associated with the Josephson junction phases. (b) A “Not”-operation in a double-well potential of the flux-driven superconducting meta-atom (α = 1.5) is shown as a transition between the states with certain values of the magnetic moment (these states correspond to the energy levels of *E*_1_ and *E*_2_, respectively). Applied adjusting magnetic flux, Φ_Z_, equal to 0.01 of the magnetic flux quantum, Φ_0_. We present here illustrations of the notations used in the text: *E*_1,_ *E*_2_, *E*_3,_ *E*_4_, Δ, Δ_2_.

In this section we develop a theoretical approach on the interaction of a multilevel superconducting meta-atom with unipolar magnetic field pulse. The considered model seems to be fairly general since it takes into account the multilevel structure of a real superconducting system and can be applied to describe the dynamics of qubit states in different types of superconducting artificial atoms. However, to be specific, we will consider the well-known flux qubit scheme: a superconducting ring with three Josephson junctions (see inset in Figure 1). The potential energy of the system takes the double-well form when the characteristic Josephson energies of the three elements are *E*_Jos_, *E*_Jos_ and α*E*_Jos_, α > 0.5. The third junction can be replaced by a superconducting quantum interferometer, which allows one to adjust α using an external magnetic flux. So, we can apply magnetic fluxes Φ*_X_* and Φ*_Z_* in order to control the barrier height and potential asymmetry respectively. Below, we show that in this way we can control the speed of transition between individual states, which is extremely important for the presented concept.

The dynamics of such a superconducting system in a magnetic pulse field is described by the non-stationary Schroedinger equation:

[1]
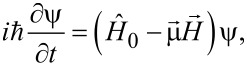


where 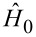 stands for the unperturbed Hamiltonian of the system, 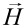 is the external magnetic field and 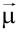 stands for the operator of the magnetic moment. In our approach we firstly take into account three energy states of such artificial Josephson atom: two qubit states with energies *E*_1,_ *E*_2_ and one upper-lying level with energy *E*_3_ (see Figure 1).

The external magnetic field 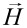 is chosen as unipolar rectangular pulse of duration τ and amplitude *A*. The key point of our approach is to tune the parameters of the meta-atom and the control circuits in order to apply the magnetic field in a proper way to block the direct transitions between the qubit states and to allow for Raman Λ-type transitions between them via the upper level. Using an existing analogy with spin states |↓⟩ and |↑⟩ the direction of 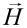 should be chosen along the z-axis such that the interaction term μ_z_*H*_z_ is proportional to the 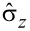 matrix, which prevents the direct spin-flip transition.

Finally we expand the non-stationary wave function of the system in terms of the considered eigenfunctions of the double-well potential φ*_n_* with energies *E_n_*,

[2]
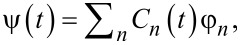


and obtain a set of coupled differential equations for the probability amplitudes *C_n_*(*t*) of the corresponding states with direct transition |φ_1_⟩ → |φ_2_⟩ being blocked:

[3]
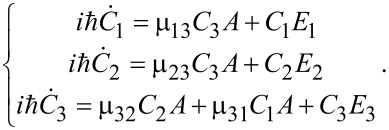


Here, μ*_ij_* are the magnetic matrix elements of the allowed transitions and for simplicity we suppose all of them to be of the same value: μ_31_ ≈ μ_32_ ≈ μ ≈ 10^6^ arb unit. Further, we solve the system (Equation 3) under the initial condition *C*_1_ = 1 which corresponds to the initial population of the lowest energy state. The time-dependent populations for any of the considered states can be calculated as follows: *W_i_* = |*C_i_*(*t*)|^2^. The obtained dynamics of the system is compared to that calculated for the case in which the transitions between all the considered states are allowed and found as solution of the following system of equations:

[4]
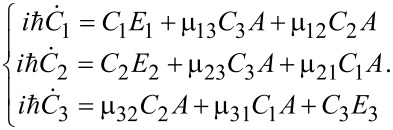


Subsequently, the time-dependent population transfer between the qubit states will be compared for both cases. Our goal is to provide an ultrafast transfer of population from one qubit state to another and to establish the regime when the population of the upper level is explicitly equal to zero because of the coherent trapping of population in the two qubit states due to the Raman Λ-type transitions induced by the magnetic field pulse.

## Results and Discussion

### Ultrafast operations in the case of blocked direct transitions between qubit states

First we analyze the possibility of the “Not”-operation induced by the unipolar magnetic pulse in the case when the direct transition between the qubit states is blocked. Under such conditions only Raman Λ-type transitions take place and are responsible for the transfer of population from the lower qubit state to the upper one. The system (Equation 3) is solved for any instant of time in the time interval [0,τ] when the magnetic field is turned on and then the field-free dynamics of the system occurs with no change of populations being observed. For convenience we introduce the following notations:

[5]
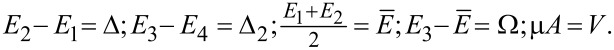


It should be noticed that since we consider a unipolar magnetic pulse which has no carrier frequency, Ω is just a designation.

Then we introduce new variables:

[6]
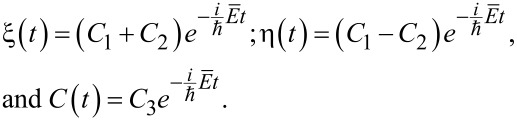


Regarding the new variables, the system (Equation 3) can be rewritten as follows:

[7]
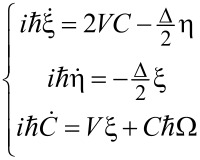


The solution of Equation 7 can be found in the form of

[8]
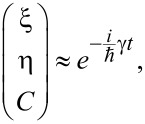


where γ is the quasi-energy of the system (Equation 7) and can be found from the equation

[9]
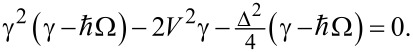


Usually for real superconducting systems Δ ≪ ℏΩ and the coupling between qubit states *V* induced by a magnetic field is rather strong due to the huge value of the magnetic dipole moment. For these reasons Equation 9 can be solved analytically by sequential iterations, and in the limit of Δ→0 the following zero-order quasi-energies are obtained:

[10]
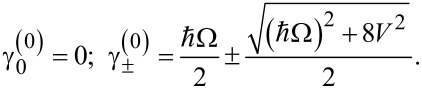


It is interesting to analyze the quasi-energy wave function 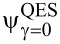 corresponding to γ^(0)^ = 0, which is found to be

[11]
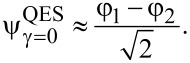


Such a wave function corresponds to the case when the whole population is trapped in the qubit states and the population of the upper state |φ_3_⟩ is equal to zero. Such a regime is referred to as a coherent population trapping known for real atomic systems in optical electromagnetic pulses [29]. If such state is chosen as the initial state, no population of the upper state |φ_3_⟩ is detected.

In this paper we consider a system being initially in the lowest state |φ_1_⟩. We focus on the possible population transfer to the qubit state |φ_2_⟩. Using the quasi-energies (Equation 10) we solve the initial value problem analytically in the limit of Δ→0 and obtain the following time-dependent amplitudes of the considered states:

[12]
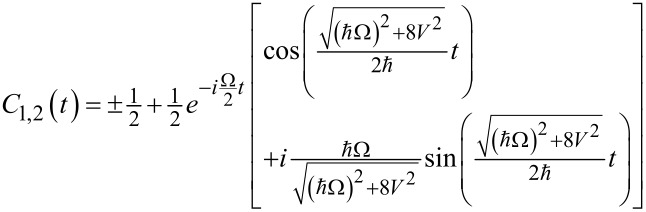


[13]
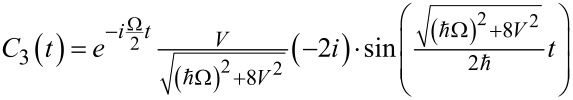


**Figure 2:**
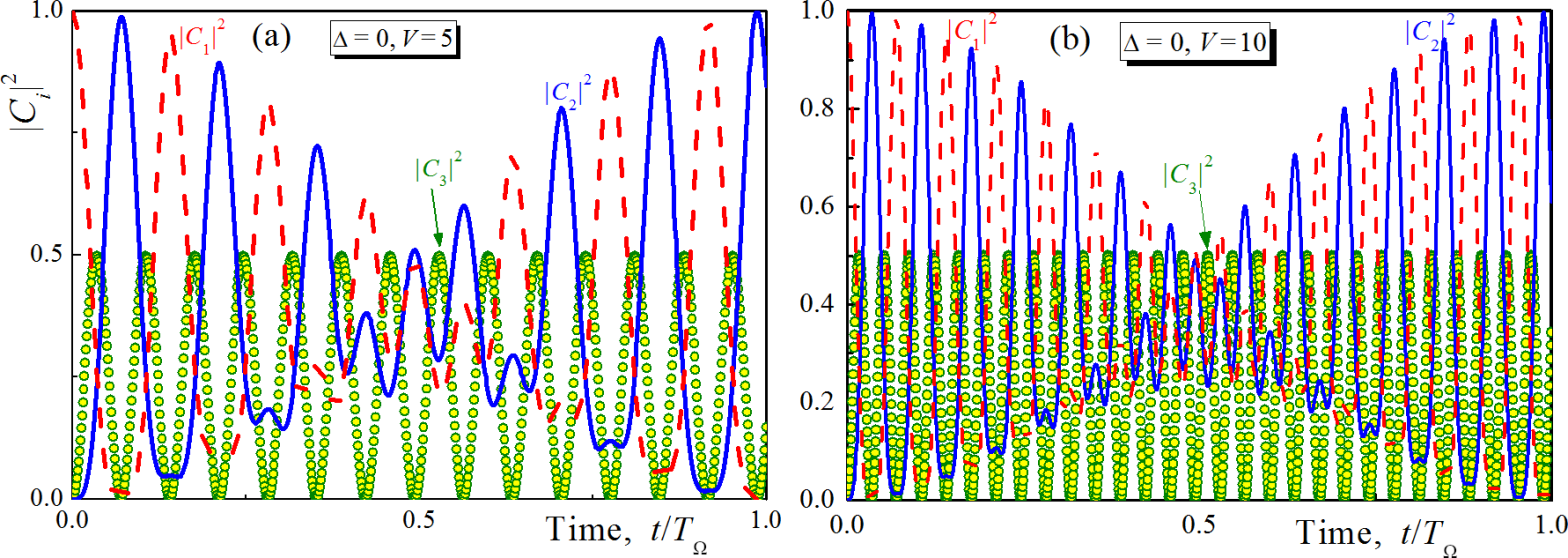
Analytical solution of Equation 7 obtained in the limit Δ→0 for two different magnetic field strengths: *V* = 5 (a) and *V* = 10 (b). Dashed lines, solid lines and circles are for |*C*_1,2,3_|^2^, respectively. The decrease of efficiency of the population transition is prevented by increasing the coupling between the qubit states. For convenience, hereinafter in the figures, the values of Δ and *V* are normalized to the characteristic energy ℏΩ.

The time-dependent populations of different states obtained from Equation 12 and Equation 13 are presented in Figure 2. They are characterized by very fast oscillations accompanied by a rather slow modulation. The fast oscillations correspond to strongly non-resonant Rabi-like transitions between each qubit state and the upper third state, which are of great efficiency due to a large value of the magnetic matrix element leading to a very strong coupling between these levels. The slow modulation represents the transition frequency Ω (or characteristic period *T*_Ω_ = 2π/Ω) between the qubit states, or rather, some mean level with energy 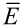 and the upper third level. It can be seen that the time of the first |φ_1_⟩→ |φ_2_⟩ transition appears to be much shorter than the period *T*_Ω_ and further decreases with a growing degree of coupling (for example, with the increase of the magnetic field strength, compare Figure 2a and Figure 2b), since the carrier oscillations become faster. As a result by increasing the magnetic field strength it is possible to provide the required operations at ultra-short time scales. For example, for μ ≈ 10^6^ arb unit, *H* ≈ 10^−5^ arb unit and Ω = 10 GHz the characteristic time of the transition between the qubit states appears to be about 5 picoseconds. This is much faster than the values of nanoseconds achieved for direct Rabi transitions.

**Figure 3:**
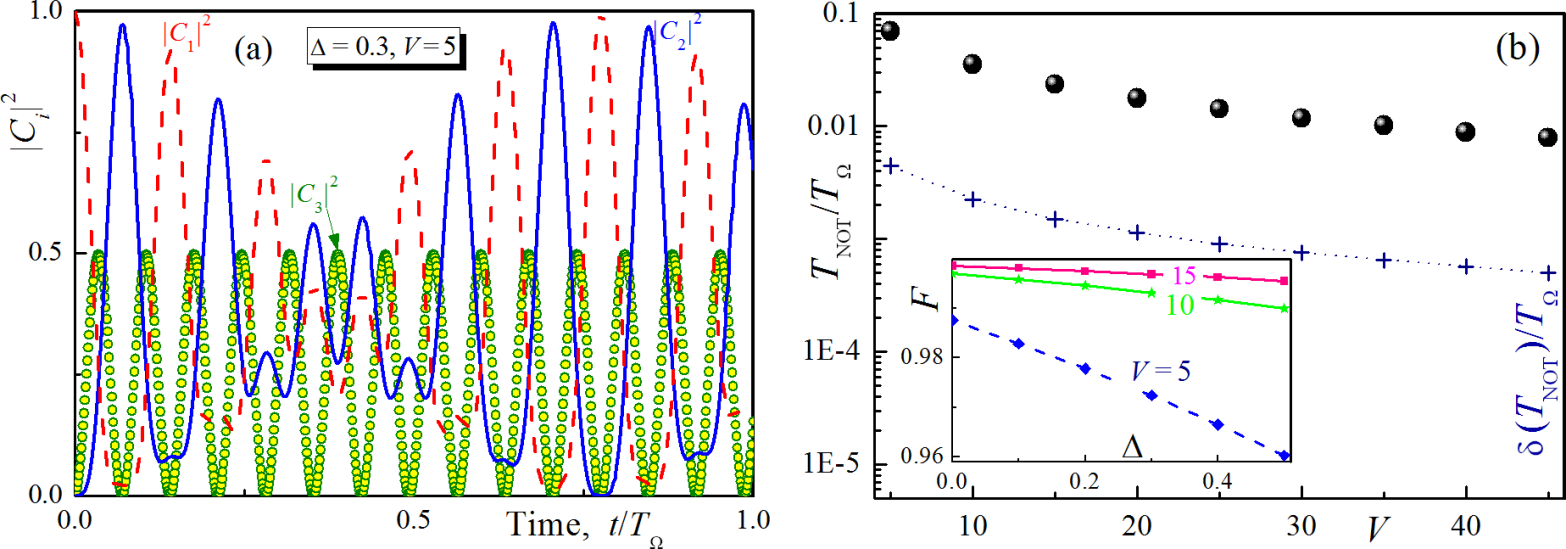
Analytical solution of Equation 7 obtained for Δ = 0.3, *V* = 5, dashed lines, solid lines, circles and squares are for |*C*_1,2,3_|^2^ (a) and the characteristic time of population transfer (dots) as well as the the required time accuracy, δ(*T*_NOT_), providing 98 percent of transition efficiency (dot line with crosses) versus the degree of coupling between the atom and the magnetic field (b). The inset in (b) shows how the efficiency of the ‘’Not”-operation operation *F* (determined by |*C*_2_|^2^ at the first maximum) decreases with increasing normalized value Δ for different coupling *V*.

Let us now discuss the dependence of the population transition time on the energy distance Δ between the qubit levels. For this goal the solution of Equation 7 was obtained using the quasi-energies found from Equation 9 by sequential iterations on the parameter Δ. The obtained analytical solution (not presented here because of its complexity) appears to be very close to the explicit numerical results. The dynamics of population of all considered levels obtained for different values of Δ is presented in Figure 3a. It can be seen that the time of the population transition is almost the same while the efficiency of transition (maximal achieved population of the second level) decreases with growing Δ (see the inset on Figure 3b). However, the efficiency can be increased by increasing the impact of the magnetic field, which provides much faster Rabi-like oscillations and, hence, almost 100% efficiency of the first peak-population transfer. Thus, while the coupling strength appears to change the carrier frequency of population dynamics, Δ is found to be responsible for the period of slow modulation.

As a result, the characteristic time of the population transition decreases dramatically with increasing coupling strength without loss of efficiency which is illustrated in Figure 3b. The time of population transfer (or “Not”-operation, *T*_NOT_) is normalized by *T*_Ω_ = 2π/Ω. While this period is in the sub-nanosecond range, the time of the considered gate appears to be up to two orders of magnitude lower. Thus we really achieve very fast gates with switching times of the order of picoseconds, which is dramatically faster than that obtained in the case of a carrier magnetic pulse resonant to the direct transition between spin states. Since in the experimental realization of such a scheme it is rather difficult to provide explicitly the required duration of the magnetic pulse, the required time accuracy providing 98% of transition efficiency is plotted in Figure 3b as a function of the atom-field coupling *V*. It can be seen that the possible deviation interval decreases with the degree of coupling. But at the same time, the characteristic time of the “Not”-operation also decreases such that the relative required time accuracy remains the same at no less than 6% of *T*_NOT_. This accuracy level seems to be achievable and can be provided in real experiments. It can be seen from Figure 2 and Figure 3 that by choosing different durations of the unipolar pulse we can rotate the Bloch vector for a qubit around the selected axis by any given angle. Together with the ability to rotate the vector around the O*_x_*- and O*_z_*-axes (e.g., due to the fluxes Φ*_X_* and Φ*_Z_*), this allows us to realize a full set of single-qubit operations through the influence of a unipolar pulse. However, in this paper we focus on the “Not”-operation gate.

### Influence of additional upper-lying levels on the ultrafast qubit control

**Figure 4:**
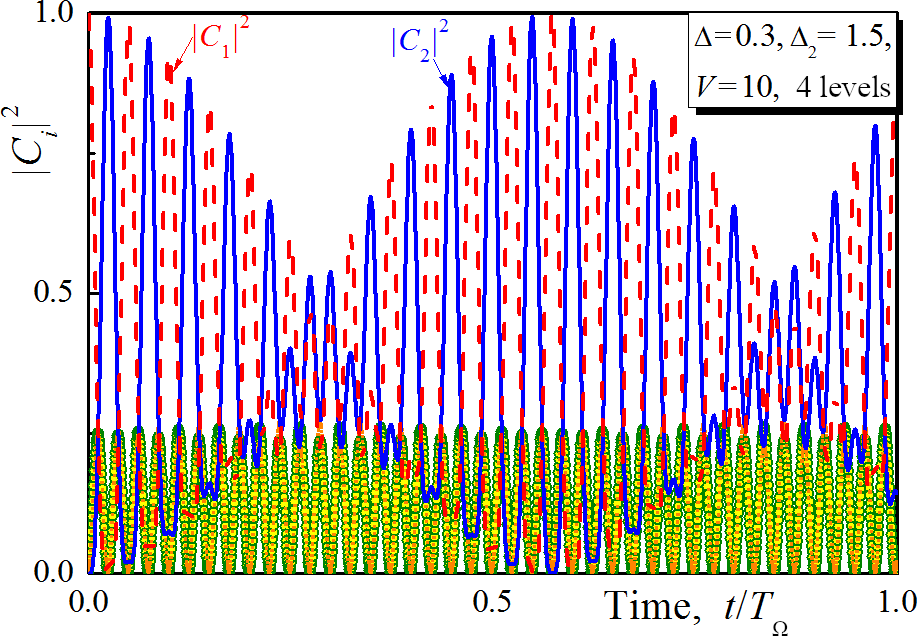
The time-dependent populations of states calculated when the 4th upper lying state of the superconducting system is involved in the Raman Λ-type transitions for Δ = 0.3, Δ_2_ = 1.5 and *V* = 10. Dashed lines, solid lines, circles and squares are for |*C*_1,2,3,4_|^2^, respectively.

Further, we have analysed the influence of the additional upper-lying levels of the studied superconducting meta-atom on the time of the obtained ultrafast population transfer. We include an additional fourth level into our considerations, and this level has a higher energy than the third one. The corresponding dynamics of population of the levels is presented in Figure 4 for the coupling parameter *V* = 10. The time of the first peak of the population transfer appears to be even shorter in comparison to the result of Figure 2b due to the increase of the frequency of slow modulations. Hence the real energy structure of the superconducting system (including even charge or hybrid qubits [1]) will not slow down the considered ultrafast control.

### Operations induced by unipolar magnetic pulse for allowed direct transitions between qubit states

Let us compare the results obtained in the previous section with the case when the direct transition between the qubit states is allowed. Such an unusual situation is possible in an artificial Josephson atom [30]. We will call it the Δ-configuration of a superconducting atom. In this case the system (Equation 4) has been solved. An analytical expression of the probability amplitude of the upper qubit state obtained in the limit of Δ→0 is given by:

[14]
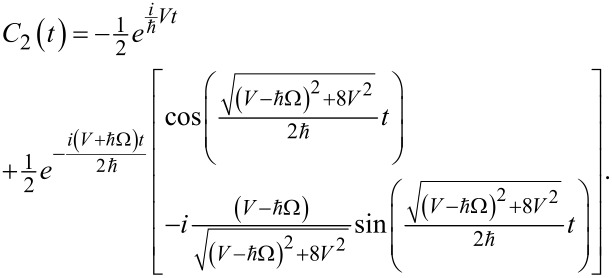


**Figure 5:**
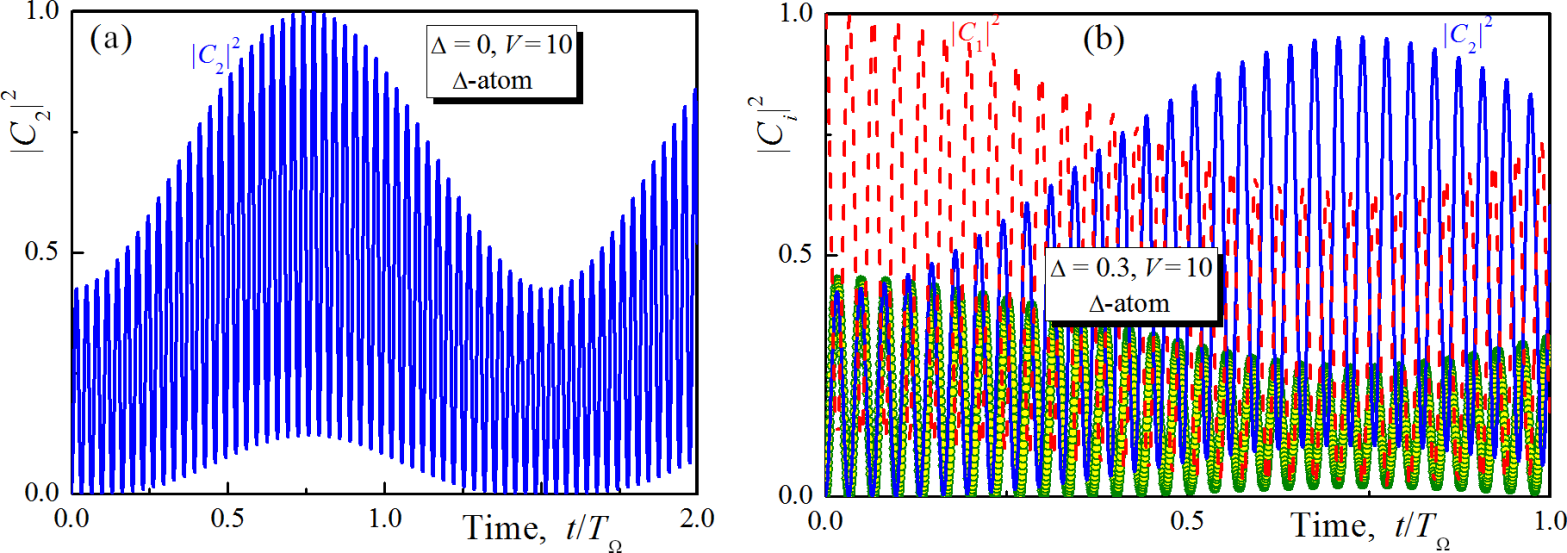
Population of the upper qubit state of the superconducting meta-atom obtained analytically for *V* = 5 in the limit of Δ→0 (a) and the populations of the all considered states calculated numerically for Δ = 0.3, *V* = 10 (b). The direct transition between the qubit states is allowed. Dashed lines, solid lines and circles are for |*C*_1,2,3_|^2^, respectively.

The corresponding dynamics of the population of this state is presented in Figure 5 for two different values of the magnetic field impact. The result is rather different from the data of Figure 2. In contrast to the case when the |φ_1_⟩→|φ_2_⟩ transition is blocked now we observe slower population dynamics with the characteristic time determined not by the separation between the qubit levels Δ, but by the period *T*_Ω_ = 2π/Ω, which corresponds to the range of nanoseconds or at least to the sub-nanosecond range for real superconducting systems. Hence, the observed population transfer appears to be significantly faster than in the case of resonant Rabi transitions but dramatically slower than in the scheme with blocked direct transition. It should be noticed that the increase of the magnetic field strength (or the coupling between levels) does not influence the characteristic time of the gate at all.

With the increase of the energy gap Δ the maximal possible value of the population of the upper qubit state starts to decrease. This means a drop of transition efficiency, while the characteristic transition time remains almost the same (see Figure 5b). Thus, in a scheme with allowed direct |φ_1_⟩→|φ_2_⟩ transitions it is not possible to speed up the population transfer significantly.

### Issues of the experimental realization of the theoretically proposed scheme

**Figure 6:**
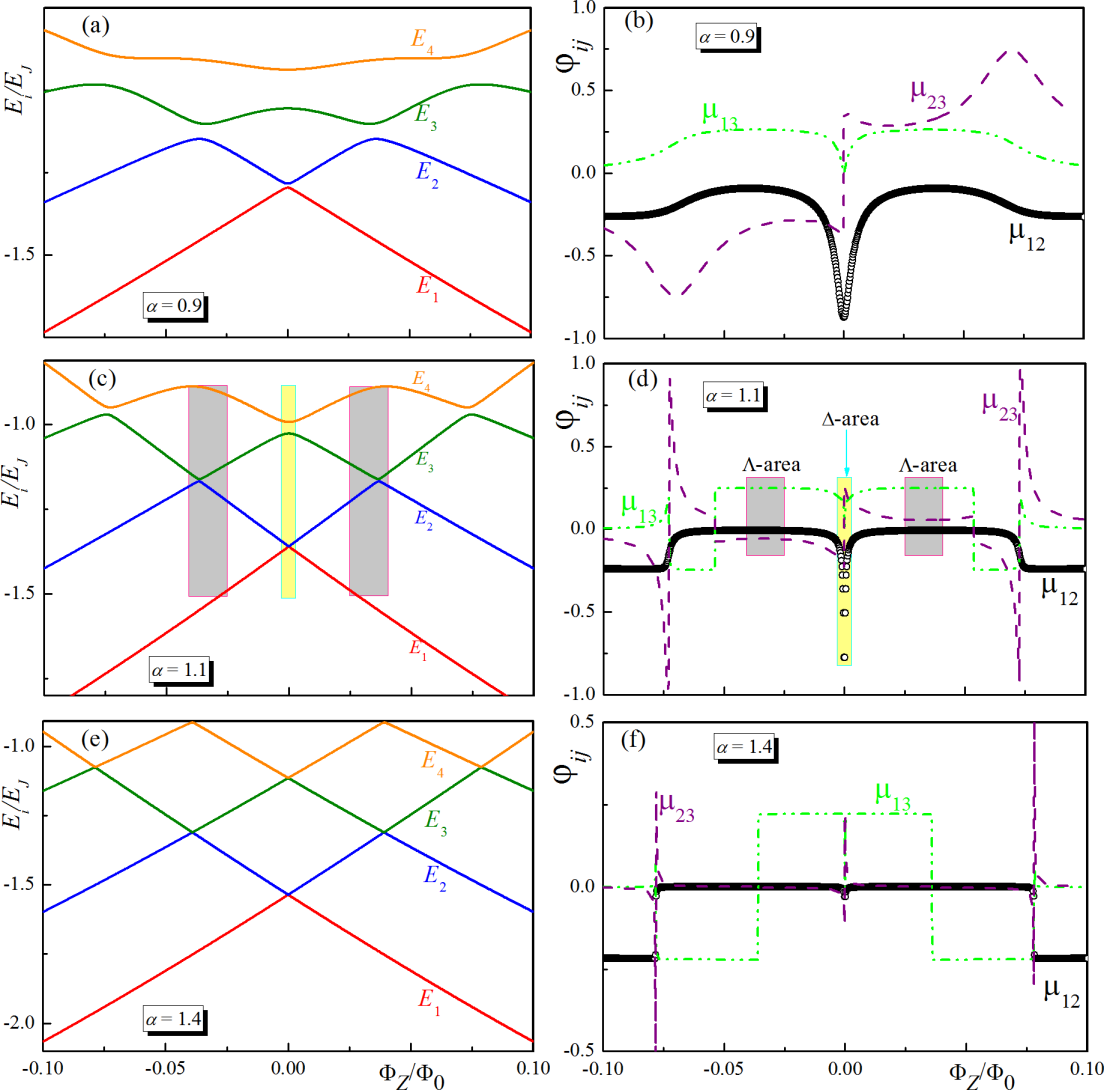
Eigenvalues, *E*_1,2,3,4_, of the unperturbed Hamiltonian and matrix elements (μ*_ij_*) for transitions between its eigenstates for different values of the potential barrier between the wells: α = 0.9 (a, b), α = 1.1 (c, d), and α = 1.4 (e,f).

First of all, we will demonstrate the possibilities for controlling the distances between energy levels and matrix elements of various transitions in superconducting meta-atoms. For this purpose, we investigated numerically eigenvalues and eigenfunctions of the unperturbed Hamiltonian 
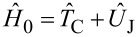
 of the system chosen earlier as an example. Here 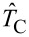 is the Coulomb energy of the system and 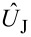 is the Josephson energy. The results of the calculations of the energy eigenvalues and the matrix elements of the operator of the generalized coordinate are presented for different values of the fluxes setting the working point (see Figure 6).

Here, the value Φ*_Z_* determines the potential asymmetry of the potential; the value Φ*_X_* determines parameter α and the size of the barrier between the minima of the potential presented in Figure 1. It can be seen that in a superconducting meta-atom we can really adjust the magnitude of the energy gap and even make it negligible. In addition, we can control the selection rules. There is a range of parameters in which, for example, the transition between the lowest energy levels is prohibited. For small sizes of the potential barrier between the minima (for example, α = 0.9), the transition between the qubit states with energies *E*_1_ and *E*_2_ is allowed over a wide range of parameters. For large barriers (α = 1.4), transitions are allowed only inside the well. In the intermediate case (α = 1–1.2) there is a mode when the |φ_1_⟩→|φ_2_⟩ transition is prohibited, but |φ_1_⟩→|φ_3_⟩ and |φ_3_⟩→|φ_2_⟩ are allowed (Λ-area). In Figure 6, we have also highlighted the areas of parameters in which all three considered transitions are allowed (Δ-area, marked in yellow).

An experimental issue of the proposed picosecond-time qubit control are the difficulties related to generation and application of the required pulses with minimal parasitic thermal perturbation of the quantum system. The first practical approach dates back to implementation of ballistic fluxon qubit readout [21–28]. The readout design was based on the well-developed rapid single flux quantum (RSFQ) circuits. Namely, a Josephson transmission line realized as a parallel array of lumped Josephson junctions coupled by superconducting inductances was chosen as a control line supporting the flux to be quantized. One cell of the transmission line was magnetically coupled with the qubit so that it was exposed to the magnetic field of the propagating fluxon for a short time. However, an experimental study presented evidence of excessive perturbation of the quantum circuits by this readout scheme.

An improved version of the classical interface was proposed in a recent work [19]. Here, the interface circuits devoted to generating and receiving control magnetic flux quanta, Φ_0_, are implemented on a different chip that is physically located at a higher temperature stage in the cryocooler. The chips with classical and quantum circuits are connected by using the standard multi-chip module (MCM) technology [31]. Control pulses are propagating in passive transmission lines coupled with qubits in the quantum part of the system. On one hand, such an approach benefits in terms of using standard digital RSFQ circuits for generating the control signal and process the response from quantum circuits while keeping them thermally isolated. On the other hand, the formation of a picosecond control pulse with appropriate parameters becomes a sophisticated problem here that requires advanced high-frequency design and was not addressed yet.

**Figure 7:**
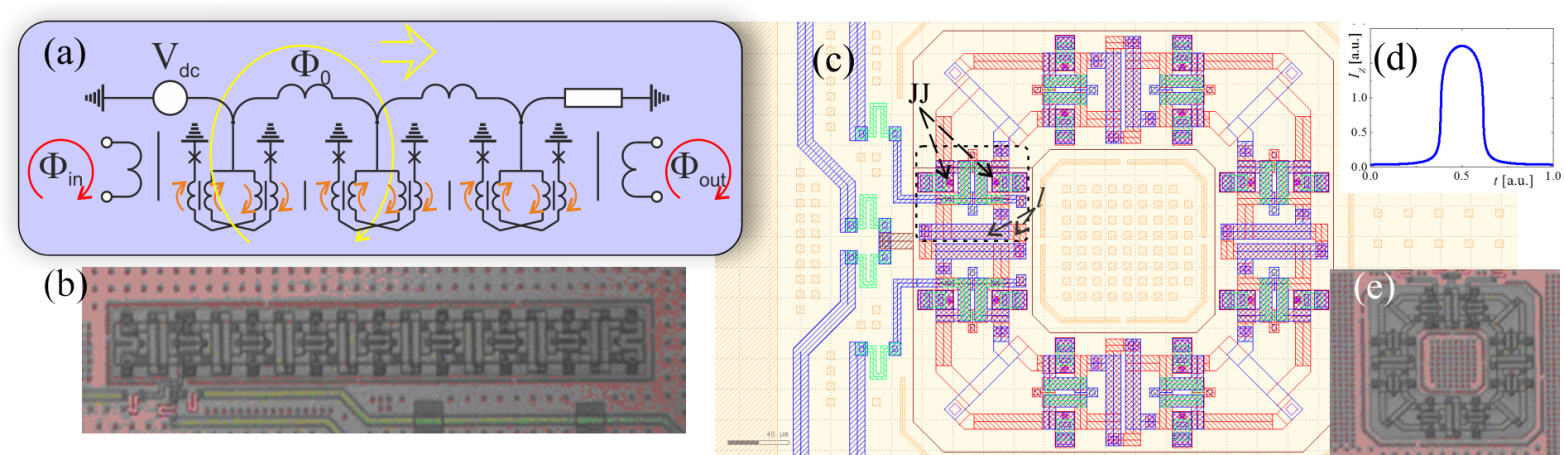
(a) A schematic diagram of a transmission line in ASL circuits, through which a soliton-like current wave can propagate, creating (from a qubit viewpoint) a control pulse of picosecond duration. Josephson junctions are depicted as crosses, the currents in the shoulder inductances are represented by short arrows. Linear (b) and circular (c, e) arrangement of elements in the experimental implementation for nSQUID-arrays manufactured by Hypres, as well as the numerically calculated current pulse in the system, the magnetic field of which affects the qubit (d). In the layout in (с), a separate ASL element is highlighted by a frame. Here, Josephson junctions and shoulder inductances are marked with “JJ” and “*l”* indices.

A simpler solution could be utilization of adiabatic superconducting logic (ASL) circuits broadly used, e.g., to control qubits in D-Wave Systems quantum processors [32]. While ASL circuits are distinguished by their ultimate energy efficiency, the shape of the magnetic signal transferred by ASL transmission line can be easily tuned in situ. In comparison with a Josephson transmission line, here Josephson junctions are substituted by superconducting adiabatic logic cells [33–36]. This allows one to transfer data not in the form of the presence or absence of quanta, but in the direction of the currents circulating in the superconducting circuits (excluding in the limit the energy dissipation due to the transition in the resistive state). Figure 7a shows schematically the information transfer in such a data bus. The role of the ASL elements here can be played by connected nSQUIDs, that is, interferometers with a negative mutual inductance of the shoulders [37–38]. The negative mutual inductance of the shoulders provides: (i) a high inductance for the bias current flowing in the same direction in both shoulders, (ii) a low inductance for the circulating current, which has different signs at the Josephson junctions. The general view and equivalent circuit of such a device are shown in Figure 7.

The sum of the Josephson and inductive energies in the system can be written through the total and difference phases, φ_±_, of the two Josephson junctions as

[15]
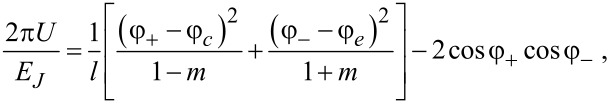


where *l* and *m* are the normalized values for the shoulder inductance (see Figure 7) and the mutual inductance correspondingly. By controlling the phases φ*_c_* and φ*_e_* (for example, using a voltage *V*_dc_, see Figure 7a), we can transfer a cell from one steady state with a certain value and direction of the circulating current to another along equipotential trajectories. This allows one to reduce decoherence in the considered scheme. Experimental studies have shown that ASL circuits demonstrate ultra-high (even for superconducting circuits) energy efficiency with an increase in operating frequencies up to several gigahertz [39]. A numerical study of the dynamic processes [40] in the nSQUID allowed us to select parameters for which it is possible for the picosecond control pulses with the required shape and accuracy to pass through the ASL transmission line. The calculated view of the revisiting control pulse for a qubit is shown in Figure 7d. Particular attention should be paid to the absence of parasitic plasma oscillations of the circuit and field in the optimal mode of operation after carrying out the required impact on the Josephson meta-atom.

## Conclusion

In this paper we develop a theoretical concept and its experimental realization of ultrafast switching between selected eigenstates of an artificial atom. The advantage of the proposed scheme consists in usage short unipolar magnetic field pulses that are characterized by a significantly broad frequency spectrum. Different spectral components of such a broadband pulse can stimulate all possible transitions in the “atomic” system. By blocking the direct transitions between selected states it is possible to controllably transfer the population between them due to Raman Λ-type transitions via upper-lying levels. As a result, the characteristic time of such “non-direct” transitions is found to be on the picosecond scale and appears to be dramatically shorter (several orders of magnitude) than the best values achieved for direct “Rabi spin-flip”. Up to now, in the Rabi-technique using carrier radio pulses with a carrier frequency of 0.1–100 GHz the achieved gate times appear to be no shorter than tens of nanoseconds due to small energy separation and near-degeneracy of the spin qubit levels [41]. This performance is limited, on the one hand, by the coherence time (which is no more than 100 μs) and by the inverse frequency of the required transition (which is 0.1–1 GHz), on the other hand. In our method the used unipolar pulse has no carrier frequency at all and the time of the induced population transfer, which is determined only by the magnetic field strength and the dipole matrix element of transition, can be significantly shortened.

Another important aspect of our suggested technique is the control over the parameters of the experimental scheme with the possibility to block the required transitions and to adjust the energy spectrum and matrix elements to their optimal values obtained from our theoretical consideration. Switching the regimes from the simple Λ-scheme to the specific case of a Δ-atom is shown and the possibility to fulfill the required conditions for the ultrafast control by unipolar picosecond pulses is demonstrated. In addition, in our experimental scheme the parameters of the magnetic field can be tuned and the optimal magnetic field strength and duration are shown to be reproduced exactly. Experiments on controlling the state of the transmon by different trains of SFQ-pulses [19–20,42] have demonstrated the fundamental applicability of superconducting digital technology in this area. Hence, the considered approach based on single unipolar field pulses and "adiabatic" variants of RSFQ circuits can also be implemented in practice, but with picosecond duration of quantum gates.

It should be emphasized that the proposed ultrafast population transfer between selected levels is important not only for the acceleration of the population transitions in superconducting qubits. It also plays a principal role in ultrafast state initialization for algorithmic quantum computers and quantum neural networks as well as in the fast control of the magnetic properties of media from Josephson meta-atoms.

## References

[1] Devoret M H, Schoelkopf R J (2013). Science.

[2] Xiang Z-L, Ashhab S, You J Q, Nori F (2013). Rev Mod Phys.

[3] van der Wal C H, ter Haar A C J, Wilhelm F K, Schouten R N, Harmans C J P M, Orlando T P, Lloyd S, Mooij J E (2000). Science.

[4] Friedman J R, Patel V, Chen W, Tolpygo S K, Lukens J E (2000). Nature.

[5] Chiorescu I, Nakamura Y, Harmans C J, Mooij J E (2003). Science.

[6] Manucharyan V E, Koch J, Glazman L I, Devoret M H (2009). Science.

[7] Steffen M, Kumar S, DiVincenzo D P, Rozen J R, Keefe G A, Rothwell M B, Ketchen M B (2010). Phys Rev Lett.

[8] Bylander J, Gustavsson S, Yan F, Yoshihara F, Harrabi K, Fitch G, Cory D G, Nakamura Y, Tsai J-S, Oliver W D (2011). Nat Phys.

[9] Grajcar M, van der Ploeg S H W, Izmalkov A, Il’ichev E, Meyer H-G, Fedorov A, Shnirman A, Schön G (2008). Nat Phys.

[10] Schoelkopf R J, Girvin S M (2008). Nature.

[11] Gu X, Kockum A F, Miranowicz A, Liu Y-x, Nori F (2017). Phys Rep.

[12] Kou A, Smith W C, Vool U, Brierley R T, Meier H, Frunzio L, Girvin S M, Glazman L I, Devoret M H (2017). Phys Rev X.

[13] Tacchino F, Macchiavello C, Gerace D, Bajoni D (2019). npj Quantum Inf.

[14] Shulga K V, Il’ichev E, Fistul M V, Besedin I S, Butz S, Astafiev O V, Hübner U, Ustinov A V (2018). Nat Commun.

[15] Hönigl-Decrinis T, Antonov I V, Shaikhaidarov R, Antonov V N, Dmitriev A Y, Astafiev O V (2018). Phys Rev A.

[16] McDermott R, Vavilov M G (2014). Phys Rev Appl.

[17] Klenov N V, Kuznetsov A V, Soloviev I I, Bakurskiy S V, Tikhonova O V (2015). Beilstein J Nanotechnol.

[18] Liebermann P J, Wilhelm F K (2016). Phys Rev Appl.

[19] McDermott R, Vavilov M G, Plourde B L T, Wilhelm F K, Liebermann P J, Mukhanov O A, Ohki T A (2018). Quantum Sci Technol.

[20] Leonard E, Beck M A, Nelson J, Christensen B G, Thorbeck T, Howington C, Opremcak A, Pechenezhskiy I V, Dodge K, Dupuis N P (2019). Phys Rev Appl.

[21] Fedorov A, Shnirman A, Schön G, Kidiyarova-Shevchenko A (2007). Phys Rev B.

[22] Herr (Kidiyarova-Shevchenko) A, Fedorov A, Shnirman A, Il’ichev E, Schön G (2007). Supercond Sci Technol.

[23] Pankratov A L, Gordeeva A V, Kuzmin L S (2012). Phys Rev Lett.

[24] Soloviev I I, Klenov N V, Pankratov A L, Il'ichev E, Kuzmin L S (2013). Phys Rev E.

[25] Fedorov K G, Shcherbakova A V, Schäfer R, Ustinov A V (2013). Appl Phys Lett.

[26] Fedorov K G, Shcherbakova A V, Wolf M J, Beckmann D, Ustinov A V (2014). Phys Rev Lett.

[27] Soloviev I I, Klenov N V, Bakurskiy S V, Pankratov A L, Kuzmin L S (2014). Appl Phys Lett.

[28] Soloviev I I, Klenov N V, Pankratov A L, Revin L S, Il'ichev E, Kuzmin L S (2015). Phys Rev B.

[29] Allen L, Eberly J H (1987). Optical Resonance and Two-level Atoms.

[30] You J Q, Nori F (2011). Nature.

[31] Gupta D, Li W, Kaplan S B, Vernik I V (2001). IEEE Trans Appl Supercond.

[32] Johnson M W, Bunyk P, Maibaum F, Tolkacheva E, Berkley A J, Chapple E M, Harris R, Johansson J, Lanting T, Perminov I (2010). Supercond Sci Technol.

[33] Takeuchi N, Ozawa D, Yamanashi Y, Yoshikawa N (2013). Supercond Sci Technol.

[34] Takeuchi N, Yamanashi Y, Yoshikawa N (2015). Supercond Sci Technol.

[35] Schegolev A E, Klenov N V, Soloviev I I, Tereshonok M V (2016). Beilstein J Nanotechnol.

[36] Soloviev I I, Schegolev A E, Klenov N V, Bakurskiy S V, Kupriyanov M Y, Tereshonok M V, Shadrin A V, Stolyarov V S, Golubov A A (2018). J Appl Phys.

[37] Ren J, Semenov V K (2011). IEEE Trans Appl Supercond.

[38] Averin D V, Xu K, Zhong Y P, Song C, Wang H, Han S (2016). Phys Rev Lett.

[39] Takeuchi N, Ortlepp T, Yamanashi Y, Yoshikawa N (2014). IEEE Trans Appl Supercond.

[40] Soloviev I I, Klenov N V, Bakurskiy S V, Kupriyanov M Y, Gudkov A L, Sidorenko A S (2017). Beilstein J Nanotechnol.

[41] Wendin G (2017). Rep Prog Phys.

[42] Howington C, Opremcak A, McDermott R, Kirichenko A, Mukhanov O A, Plourde B L T (2019). IEEE Trans Appl Supercond.

